# Prevalence of coat colour traits and congenital disorders of South American camelids in Austria, Germany and Switzerland

**DOI:** 10.1186/s13028-020-00554-y

**Published:** 2020-09-18

**Authors:** Stéphanie Mali Jost, Andrea Knoll, Gesine Lühken, Cord Drögemüller, Patrik Zanolari

**Affiliations:** 1 Clinic for Ruminants, Vetsuisse Faculty, University of Bern, Bremgartenstr.109a, Bern, 3001 Switzerland; 2 Institute of Genetics, Vetsuisse Faculty, University of Bern, Bremgartenstr.109a, Bern, 3001 Switzerland; 3 Institute of Animal Breeding and Genetics, Justus Liebig University, Liebigstr. 21, Giessen, 35390 Germany

**Keywords:** Alpaca, Coat colour, Coat pattern, Congenital disorder, Fleece, Llama, Survey

## Abstract

**Background:**

The increasing popularity of alpacas and llamas outside of South America is undeniable. The associated limited genetic diversity raises questions about health and other genetically determined traits like coat colour. Therefore, a survey studying the prevalence of congenital disorders and coat colours and patterns in South American camelids was performed in Austria, Germany and Switzerland. Moreover, the motivation for keeping these animals, the herd size and breeds was assessed.

**Results:**

A total of 146 questionnaires were returned corresponding to 16 farms from Austria, 69 farms from Germany, and 61 farms from Switzerland. In total, the returned surveys reported data on 2770 animals including ~ 85% alpacas and ~ 15% llamas. The most common alpaca breed was *Huacaya* (87.7%), the most common llama breed was *Wooly* (15.6%). Breeding (69.4%), wool production (63.3%) and keeping them as pets (53.7%) were the most common motivations to keep these animals, although this varied among countries. The three coat colour groups, solid white (24.8%), brown and black (64.8%) and grey (10.4%), occurred at different frequencies. About 7% of the South American camelids with solid white coat showed blue-pigmented eyes, corresponding to the known blue-eyed white phenotype, of which more than every second animal was apparently deaf. Uniform solid coloured animals occurred predominantly (81.4%), whereas pinto (8.8%), speckled (6.4%) and spotted (3.4%), also known as appaloosa, were comparably less prevalent. In total 161 observations of congenital disorders occurring during a 5-year-period were reported. The most prevalent disorders were in the group of musculoskeletal disorders such as spiral toe growth (16.4%), hyperextension of the fetlock joint (12.3%), angular limb deformities (11.0%) and axial rotation of the limbs (8.2%).

**Conclusions:**

This survey revealed first insights into the occurrence of different traits and disorders in the current South American camelid population of Austria, Germany, and Switzerland. The identification of the most common musculoskeletal disorders might encourage the breeders to eliminate affected animals from their breeding program to decrease the incidence although traits such as spiral toe growth might also represent phenocopies.

## Background

The two domestic South American camelids (SACs) species, the alpaca (*Vicugna pacos*) and the llama (*Llama glama*), have become increasingly popular in the last few decades in regions outside South America such as the USA, Australia and Europe [1]. In general, in these regions, alpacas are more popular and are sometimes kept together with llamas [2]. An increasing number of veterinarians now find SACs under their care. However, there is little published information e.g. about the health status of the SAC populations. A first survey of the health of SACs in the United Kingdom provides information on population statistics, mortality rates and possible causes of death [1] whereas a second questionnaire focussing on reproduction traits revealed that a third of mated female SACs failed to produce offspring [3]. In Switzerland, results of a first survey published in 2005 showed that 999 (61.6%) of the Swiss SAC population consisted of llamas and 623 (38.4%) of alpacas and that they were predominantly kept as pets or for breeding or trekking whereas the most frequent health problems were related to the digestive tract, the skin, the eyes and the metabolism [4]. More recently, a Swedish study revealed the number of deaths from emaciation in weanling alpacas during late winter or early spring and infectious and non-infectious causes of death in adult alpacas [5].

In Europe, SACs are predominantly bred by natural service, as advanced reproductive techniques are not routinely used [6, 7]. In addition, today’s SACs outside of South America are descended from a limited number of animals and the need to import living animals instead of frozen semen or embryos is limiting the increase of genetic diversity. Furthermore, the breeders focus on economically important traits such as fleece quality and coat colour, and thus are intensively using only specific promising males and their progeny for breeding. Altogether, this favours inbreeding that can lead to the emergence of inherited disorders. For example, due to the observation of an increased number of ocular diseases being reported in SACs it was speculated that this could be the result of inbreeding [8]. Therefore evaluating the genetic health of SAC populations outside of their continent of origin is particularly interesting and may be of value for both SAC breeders and veterinary practitioners advising them about breeding programs in future.

Currently, the Online Mendelian Inheritance in Animals (OMIA) database of inherited disorders contains 11 and 17 entries including coat colour-associated disorders for alpacas and llamas, respectively [9]. For instance, there were cases of choanal atresia, hernias, angular limb deformities, polydactyly, cleft palate, atresia vulvi, ventricular septal defect, hypoplasia of the reproductive organs, congenital cataract, bilateral deafness in animals with pure white coat and blue iris pigmentation, ocular abnormalities and nasolacrimal duct aplasia were reported in single case studies [10–22]. The most common diagnosis among 56 sick juvenile individuals (crias) was the presence of at least one congenital disorder such as choanal atresia, ocular malformation, bone deformation, vulvar deformation, hermaphroditism, or cardiac malformation [23]. For most of these congenital disorders observed in SACs the aetiology is unknown and a genetic cause is often highly suggested. For example, choanal atresia (OMIA 001764-30538) is a common nasal craniofacial malformation that resembles human CHARGE syndrome (OMIM 214800) but so far, a pathogenic variant in the associated *CHD7* gene has not yet been found in affected alpacas [24]. Taken together, this illustrates the need to evaluate the prevalence of congenital disorders in SACs.

Beside rare disorders, fleece quality and coat colour are economically important genetic traits in SACs. The most important fleece types and the source of the name of the two most common alpaca breeds are *Huacaya* and *Suri* (OMIA 001528-30538). The *Huacaya* type resembles the wool of Merino sheep whereas the *Suri* type has silkier fibres in “cork screw” shaped locks. It was suggested that the *Huacaya* phenotype is caused by homozygosity of recessive alleles at two linked loci [25]. The three known llama breeds *Classic*, *Wooly* and *Suri*, although they are not bred as intensively for their fleece as alpacas, are also distinguished by their fleece quality. Although the coat colour of alpacas and llamas, especially the white ones, is essential for the textile industry, the genetic basis of coat colours is mostly unknown. The *KIT* gene has been postulated as candidate for the dominantly inherited white phenotype in alpacas [26] but despite strong association, no unequivocal relationship between the blue-eyed white (BEW) phenotype and *KIT* genotype was revealed [27]. Recent data provides evidence that the dominant white or light grey phenotype of alpacas is caused by a *KIT* missense variant that is most likely lethal in homozygous state [28]. The classic grey phenotype is a characteristic dilution of the base colour and can occur over a eumelanic (silvergrey) or pheomelanic (rosegrey) base colour due to unknown mechanisms that govern the variable intensity [28]. In addition, the *KRT2* and *ASIP* genes seem to influence colour determination of alpacas [29, 30] whereas the *MC1R* gene might influence coat lightness [31]. For llamas it was shown that the expression of *KIT* and *MITF* was significantly lower in white animals than in coloured ones [32].

This study presents the results of a survey sent to all members of the SAC breeders’ associations of Austria, Germany and Switzerland and then evaluated in order to estimate the occurrence of coat colour traits and congenital disorders in alpacas and llamas.

## Methods

In Switzerland an online questionnaire in both French and German was created, which was given access by an URL-link (Additional file 1). This link was published on the homepage of the national SAC association and the members were informed by e-mail. To attract attention, an article was posted in the Swiss magazine Forum für Kleinwiederkäuer for small ruminants and South American camelid farmers and in addition the survey was promoted (inter-) national conferences. In Germany and Austria, the URL link for the questionnaire with similar content was sent to the different breeding associations. The associations were asked to forward this to their members. The links were opened on March 29th, 2019 and all answers received by May 31st, 2019 were included in the results. Due to this procedure, the exact number of recipients is unknown.

The questionnaire consisted of three parts. The aim of the first part was to identify species and breeds, whereas multiple answers were allowed, the number of animals and since when and why the SACs had been kept on the farms. The second part aimed to get data on the current occurrence of the 16 natural coat colours internationally used by the breeding associations [33] and the 4 different coat patterns (uniform, pinto, speckled, spotted/appaloosa). If an animal was multi-coloured, it would count for each of its colour. In case of solid white animals with blue eyes it was additionally asked, if they were deaf.

The last section of the questionnaire listed congenital disorders. In general, the owners were asked if they had seen the listed disorders over the last 5 years (2014–2019), as well as if they had noticed animals that had more than one anomaly at a time. This list contained 28 disorders reported to occur in SACs in the scientific literature as well as further abnormal phenotypes to be included in the survey by Swiss farmers. The survey contained a short, illustrated description of disorders, which were divided into five different anatomical or functional groups (head area, musculoskeletal, reproduction, behaviour, and other). The assignment of disorders to groups is given in Additional file 2. Finally, the survey allowed to add comments or unlisted congenital phenotypes.

## Results

A total of 146 questionnaires were returned corresponding to 16 farms from Austria, 69 farms from Germany, and 61 farms from Switzerland. In total, the returned surveys reported on 2770 animals including 225 alpacas from Austria, 1506 (90.2%) alpacas and 163 (8.8%) llamas from Germany, and 614 (70.1%) alpacas and 262 (29.9%) llamas from Switzerland. Of the 146 participating farms, 78.8% kept only alpacas, 10.8% only llamas, whereas 10.2% of the farms kept animals of both species. Among the alpacas *Huacaya* (87.7%) dominated over the *Suri* (21.8%), whereas 15.6% of the farms have *Wooly* llamas, *Classic* llamas 12,2%, and 6.8% *Suri* llamas.

**Fig. 1:**
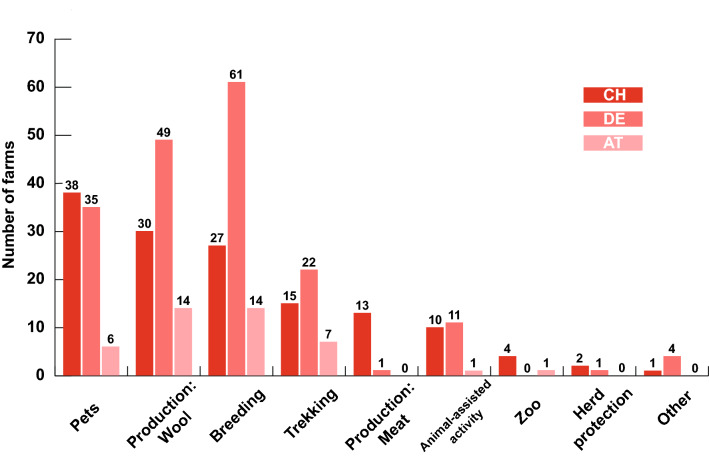
Motivation of farmers to keep South American camelids. (*AT* Austria, *DE* Germany; *CH* Switzerland)

The three most common motivations were to breed animals (69.4%), followed by wool production (63.3%), and keeping SACs as pets (53.7%). In addition, other three less often mentioned motivations were trekking (29.9%), animal-assisted activities (15.0%), meat production (9.5%), zoo (3.4%) and herd protection (2.0%). These motivations differed between countries (Fig. 1): e.g. in Austria breeding of animals and production of wool occurred at the same frequency (14 out of 16 farms each), in Germany breeding was the strongest motivation (61 out of 69 farms) whereas in Switzerland the most often frequently given reason was keeping them as pets (38 out of 61 farms).

### Coat colour traits

**Fig. 2:**
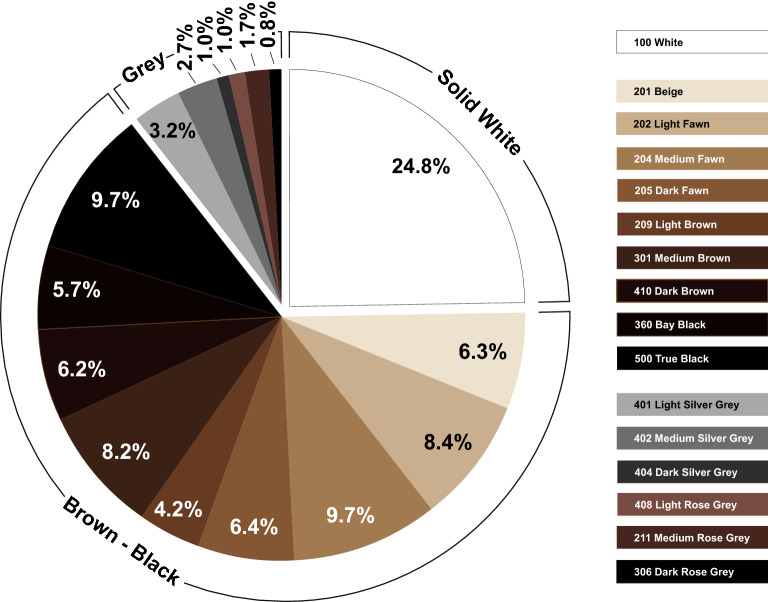
Reported coat colours in South American camelids. The survey was based on the 16 natural coat colours internationally used by the breeding associations shown on the right. Note that the three base colour groups white, brown and grey occur at different frequencies

**Fig. 3:**
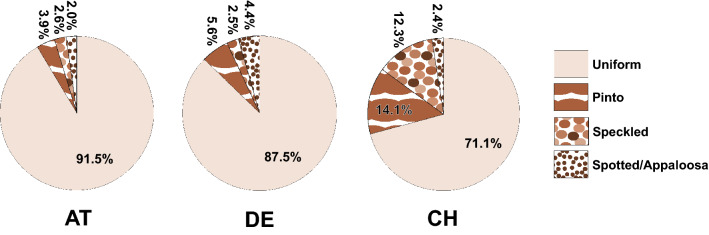
Reported coat patterns in South American camelids in Austria, Germany and Switzerland. The survey was based on the four natural coat patterns internationally used by the breeding associations shown on the right. Note due to rounding the frequencies do not sum up to 100%

Based on the returned answers, the occurrence of the different coat colours can be divided into three groups: solid white, brown/black and grey colours (Fig. 2). The solid white coat colour termed W 100, although at a different level, is with 24.8% by far the most common base colour in all three countries. Interestingly, 7.2% of these solid white animals were reported to show blue-pigmented eyes, corresponding to the known blue-eyed white (BEW) phenotype. Among the BEW animals, more than every second (53.3%) was suspected as deaf. Within the most prevalent group (64.8%) of coat colour containing nine shades of brown and black, the different colours occurred in frequencies between 3.8 to 8.7% (Fig. 2). Compared to the group of brown and black coat colours, the six grey coat colours occur more rarely (Fig. 2). The individual grey colours appear at different frequencies from 0.8 to 3.2%. Interestingly, the rarest colour dark rose grey (306 DRG) occured in all three countries, whereas the colour medium silver grey (402 MSG) was reported only by Swiss breeders. Solid colouring, which means the animal’s whole body is of one colour, is by far the most common (Fig. 3). Other coat pattern or depigmentation types are comparably less prevalent. In detail, the three different non-solid coat patterns are more often found in Switzerland than in Germany and Austria with pinto 14.1% and speckled 12.3% (Fig. 3).

### Congenital disorders

**Fig. 4:**
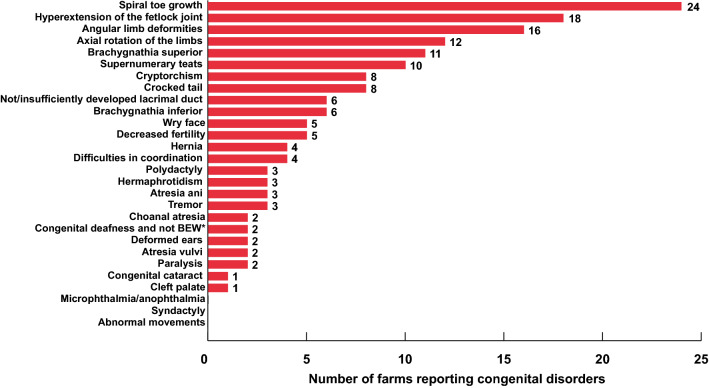
Occurrence of farms reporting congenital disorders of South American camelids during a 5-year-period (2014-2019). *BEW blue-eyed white

Across the three countries, 161 observations of congenital disorders occurring during the 5-year-period were reported (Additional file 2). Even though there were slightly more farms from Germany participating in this study, Swiss farmers reported 98 observations of congenital disorders during the last 5 years, whereas only 60 observations were noticed in Germany during that period. The numbers of reported congenital defects were even smaller in Austria: only three out of the sixteen participating farmers in Austria reported to have observed congenital disorders. For 25 out of the 28 enquired disorders, a different number of observations ranging from 1 to 24 was made (Fig. 4). The eight by far most prevalent defects were spiral toe growth (16.4%), followed by hyperextension of the fetlock joint (12.3%), angular limb deformities (11.0%), axial rotation of the limbs (8.2%), brachygnathia superior (7.5%), supernumerary teats (6.8%), cryptorchism and crocked tail (5.5% each) (Additional file 2). Further 10 disorders occurred less frequently: insufficiently or no developed lacrimal duct (4.1%), brachygnathia inferior (4.1%), wry face (3.4%), decreased fertility (3.4%), hernias (2.7%), difficulties in coordination (2.7%), polydactyly (2.1%), hermaphrotidism (2.1%), atresia ani (2.1%), tremor (2.1%). Five congenital disorders were observed only twice (choanal atresia, non-BEW-associated deafness, deformed ears, atresia vulvi, and paralysis) and two congenital disorders (cataract and cleft palate) were observed only once, whereas microphthalmia/anopthalmia, syndactyly, and abnormal movements were not reported.

## Discussion

Animal health surveys at population and herd levels are an appropriate tool as an essential first step in identifying priorities for research and formulating hypotheses for more detailed analytical studies. As there are no publications on the genetic health of animals in the current SAC population of Austria, Germany and Switzerland, initially an informal survey was carried out to obtain first information. The animals of these countries are often seen as one population as trading and breeding across borders is common practice. This survey focussing on different coat colour and pattern plus congenital disorders in alpaca and llamas revealed first insights into their occurrences.

The level of details in the present study is limited by its design, especially as SAC owners had to remember rare cases of congenital disorders in the course of the past five years. Often, the cause of a single case remains unnoticed or forgotten. On the one hand, professional breeders probably pay more attention to congenital disorders than regular SAC keepers. On the other hand, the obvious differences between the countries indicate reliability in answering the questionnaire. Nonetheless, we cannot rule out that the survey was biased.

First of all, the obtained data showed an increase of the number of farms keeping SACs over the last two decades. This obvious recent increase of farms keeping SACs observed in all three participating countries reflects the growing popularity of SACs due to various reasons. Interestingly, regarding the motivation for keeping SACs, a survey performed in Switzerland 20 years ago obtained very similar results [4]. In detail, only a few farmers (6.7%) have had SACs for more than 20 years; approximately one-third of farmers began keeping SACs between 2000 and 2009, whereas most (62.2%) started within the last decade. In contrast, in Switzerland the official statistic shows that the total number of SACs has been slightly decreasing for three years now [34]. Unfortunately, there is no national census of camelid owners neither in Austria nor in Germany.

The obtained results of this survey are predominantly about alpacas. Obviously, there are relatively more alpacas kept today compared to data obtained 20 years ago [4]. Actually, in 2017 in Switzerland 3666 alpacas and 2953 llamas were officially recorded [34]. Neither in Germany nor in Austria exact statistics of the number of SACs are available. The animals included in the questionnaires from Switzerland correspond to 13.2% of the Swiss SAC population, namely 16.7% of the alpacas and 8.8% of the llamas. The smaller proportion of answers obtained for llamas is possibly due to the lower number of animals of that species in Austria and Germany. It could be speculated that llamas probably were kept less frequently there due to their lower economic value for fibre production.

In general, solid white seems to be by far the most popular coat colour. Seven percent of these solid white animals were BEWs, and every other animal was apparently deaf. This ratio seems to be slightly lower as reported before. In a clinical study, seven out of nine alpacas with solid white coats and bilaterally blue irides were diagnosed deaf using brainstem auditory-evoked potential test [19]. Interestingly, in comparison to farmers in Austria and Germany Swiss farmers prefer white (non-pigmented) animals less whereas the non-solid (spotted) coat patterns occur more often in Switzerland. In addition, instead of keeping solid white animals the Swiss farmers prefer the four darker coat colours medium brown (301 MB), dark brown (410 DB), bay black (360 BB) and true black (500 TB). The majority of the Swiss farmers keep SACs as pets and, presumably, were not breeding for economically valuable traits like wool, but rather for aesthetic properties and personality traits. However, in the textile industry the white wool is the most preferred. Therefore, the global alpaca industry currently pays significantly more for white fleece, as it can be dyed to any desired colour [35]. The removal of the chemical dying process for certain colours would offer considerable cost savings while also presenting a sustainable alternative to the market [36]. This means the uniform pure white alpaca or llama is the most economically valuable animal for wool production, which reflects the mentioned motivation of keeping these animals especially in Austria and Germany. It is conspicuous that the group of grey colours occurs comparatively rare (10.4%). This may be due to the fact that classical grey is linked with embryonal mortality and may also be associated with the BEW phenotype [28]. Therefore, a lower number of animals with grey colours may be produced because mating classical grey animals with each other is avoided by most breeders or, if not avoided, results in a lower number of progenies.

The most prevalent congenital disorders reported within the studied SAC populations belong to the musculoskeletal group. Nonetheless, further e.g. environmental factors causing phenocopies, which are incidents in which non-genetic conditions simulate a genetic disorder, have to be taken into account as well e.g. the most prevalent spiral toe growth can also occur due to lack of claw trimming. Particularly, limb anomalies such as spiral toe growth, hyperextension of the fetlock joint and axial rotation of the limbs occurred most frequently. The latter three traits were added to the questionnaire as recommended by the Swiss SAC breeders, although yet, to the knowledge of the authors, no scientific report indicated the importance of these disorders. The observed high prevalence of these defects in all three countries could be explained either by the high awareness in the community or because these disorders are less severe than others and the reluctance to breed with affected animals is low. In contrast to a study from the USA [23], the choanal atresia was observed only two times. Therefore, it obviously not belongs to the most common congenital defects in the studied SAC population. Choanal atresia is suggested to be a monogenic Mendelian trait [24] and this congenital disorder is surgically correctable, but to our knowledge this surgery is not as routinely performed in Europe as in the USAa. This could explain that affected animals are more frequently excluded from active breeding in Austria, Germany and Switzerland.

## Conclusions

Various coat colours and different patterns occurred with regional differences whereas a quarter of the animals showed the solid white coat colour. Deafness and choanal atresia occurred less frequently compared to previous reports. The most frequently reported congenital disorders are musculoskeletal anomalies such as spiral toe growth, hyperextension of the fetlock joint, axial rotation of the limbs and angular limb deformities. These results might encourage the breeders to eliminate affected animals from their breeding program to decrease the incidence. The efficiency of this could be improved if research will be undertaken in order to develop gene tests for selection.

## Supplementary information

**Additional file 1:** Original questionnaire (version used in Switzerland) translated into English.

**Additional file 2:** Reported congenital disorders from South American camelid farms in Austria (AT), Germany (DE) and Switzerland (CH) during a 5-year-period (2014–2019).

## Data Availability

The datasets used and/or analysed during the current study are available from the corresponding author on reasonable request.

## Abbreviations

ASIP: Agouti signal peptide
BEW: Blue-eyed white
CHD7: Chromodomain helicase DNA binding protein 7
KIT: KIT proto-oncogene, receptor tyrosine kinase
KRT2: Keratin 2
MC1R: Melanocortin 1 receptor
MITF: Melanocyte inducing transcription factor
SAC: South American camelid
