# Supplementary material for: Prevalence of coat colour traits and congenital disorders of South American camelids in Austria, Germany and Switzerland
Source: Acta Vet Scand. 2020 Sep 18;62:56. doi: 10.1186/s13028-020-00554-y (PMC7501662; doi:10.1186/s13028-020-00554-y)
Supplement: Supplementary file 1 — Additional file 1: Original questionnaire (version used in Switzerland) translated into English. [file 13028_2020_554_MOESM1_ESM.pdf]

Verein der
Neuweltkameliden
Schweiz

Beratungs- und
Gesundheitsdienst für
Kleinwiederkäuer

UNIVERSITÄT
BERN

Vetsuisse Faculty
Clinic for Ruminants
Institute of Genetics

Prevalence of coat colour traits and congenital disorders of South American camelids

To whom it may concern,

within a master thesis we are investigating which genetic traits are present in South American Camelids. This includes, for example, investigating the heredity of the different coat colours, but also to find out which anomalies occur sporadically or even more frequently in our llamas and alpacas. In cooperation with the Beratungs- und Gesundheitsdienst für Kleinwiederkäuer (Advisory and Health Service for Small Ruminants) and the Verein der Neuweltkameliden Schweiz (Swiss Association of South American Camelids) we created a questionnaire.

We kindly ask you to fill in the questionnaire by 31st May 2019

Thank you very much for your participation!

Stéphanie Jost, Master Student of Veterinary Medicine, Vetsuisse Faculty, University of Bern
Patrik Zanolari, PD Dr. med. vet., Clinic of Ruminants, University of Bern
Cord Drögemüller, Prof. Dr. med. vet., Institute of Genetics, University of Bern

b
UNIVERSITÄT
BERN

1. General

1.1 Which animals do you keep?

- ☐ Llama
- ☐ Alpaca
- ☐ both

1.2 How many of these animals do you keep?

____

1.3 Which breeds do you keep?

- ☐ Huacaya Alpacas
- ☐ Suri Alpacas
- ☐ Classic Llamas
- ☐ Wooly Llamas
- ☐ Suri Llamas
- ☐ other ____________

1.4 What is your motivation to keep these animals? Multiple answers are possible

- ☐ Hobby
- ☐ Trekking
- ☐ Breeding
- ☐ Production: Meat
- ☐ Production: Wool
- ☐ Herd protection
- ☐ Zoo
- ☐ Animal-assisted activities
- ☐ other ____________

1.5 Since when do you keep South American Camelids? Please state the year.

____

2. Coat traits

2.1 Which coat colours do your animals have? Please indicate the number of animals which have this coat colour. If an animal is multicoloured, you can list the animal several times.

- ☐ Blue-eyed white ____
  - o If yes: are/were these animals deaf?
    ☐ Yes ☐ No
- ☐ 100 W White ____
- ☐ 201 B Beige ____
- ☐ 202 LF Light Fawn ____
- ☐ 204 MF Medium Fawn ____
- ☐ 205 DF Dark Fawn ____
- ☐ 209 LB Light Brown ____
- ☐ 301 MB Medium Brown ____
- ☐ 410 DB Dark Brown ____
- ☐ 360 BB Bay Black ____
- ☐ 500 TB True Black ____
- ☐ 401 LSG Light Silver Gray ____
- ☐ 402 MSG Medium Silver Gray ____
- ☐ 404 DSG Dark Silver Gray ____
- ☐ 408 LRG Light Rose Gray ____
- ☐ 211 MRG Medium Rose Gray ____
- ☐ 306 DRG Dark Rose Gray ____

2.2 Which coat patterns do your animals have? Please indicate the number of animals which have those coat pattern.

☐ Uni	(only one colour)	____
☐ Pinto	(two or more colours on a large surface)	____
☐ Speckled	(two or more colours on a small surface)	____
☐ Appaloosa/Spotted	(two or more colours, spots)	____
☐ Other __________________		____

3. Anomalies

3.1 Have you observed the following anomalies in your animals in the last 5 years?

Head Area

- Choanal Atresia
- „Wry face“
- Cleft palate
- Congenital cataract
- Not/insufficiently developed lacrimal duct
- Microphthalmia or no eyes at all
  Congenital deafness and not Blue-eyed white
- Deformed ears
- Brachygnathia inferior
- Brachygnathia superior

Muskuloskeletal

- Angular limb deformities
- Polydactyly
- Syndactyly
- Hyperextension of the fetlock joint
- Axial rotation of the limbs
- Spiral toe growth

Reproduction

- Cryptorchism
- Atresia vulvi
- Hermaphrotidism
- Supernumerary teats

  If yes: were they producing milk?

   Yes No
- Increased infertility
  - o If yes: Please comment on how you experienced the infertility: _______________

Other

- Atresia ani
- Hernia
- Crocked tail

3.2 Have you had animals showing several of these deformities in the last 5 years?

- ☐ Yes. Which ones? ________________

- ☐ No

3.3 Have you observed malformations in the last 5 years which are not listed here?

____

4. In conclusion

4.1 In case of a follow-up study: would you be willing to make your animals available for more detailed genetic analyses?

- ☐ Yes (please fill in the address field completely)
- ☐ No

4.2 Do you want to be informed about the further course of the project and the final evaluation?

- ☐ Yes (please enter your E-Mail)
- ☐ No

Your contact details

Name ________________

Address ________________

Postcode, City ________________

E-Mail ________________

Phone Number ________________

Information on data protection

All personal data will be treated confidentially and will not be passed on to third parties. Your data will only be used in the context of this master thesis. The results of the study can be published, but it will not be possible to draw conclusions about the identity of the participating person.
