# Supplementary material for: Prevalence of coat colour traits and congenital disorders of South American camelids in Austria, Germany and Switzerland
Source: Acta Vet Scand. 2020 Sep 18;62:56. doi: 10.1186/s13028-020-00554-y (PMC7501662; doi:10.1186/s13028-020-00554-y)
Supplement: Supplementary file 2 — Additional file 2: Reported congenital disorders from South American camelid farms in Austria (AT), Germany (DE) and Switzerland (CH) during a 5-year-period (2014–2019). [file 13028_2020_554_MOESM2_ESM.pdf]

Additional file 2. Reported congenital disorders from South American camelid farms in Austria (AT), Germany (DE) and Switzerland (CH) during a 5-year-period (2014–2019).

Group	Congenital Disorder	AT (n=16)	DE (n=69)	CH (n=61)	Total (n=146)	Prevalence (%)
Head area	Brachygnathia superior		3	8	11	7.5
	Not/insufficiently developed lacrimal duct		3	3	6	4.1
	Brachygnathia inferior		5	1	6	4.1
	Wry face		3	2	5	3.4
	Choanal atresia		1	1	2	1.4
	Congenital deafness and not BEW*		1	1	2	1.4
	Deformed ears		2		2	1.4
	Congenital cataract			1	1	0.7
	Cleft palate		1		1	0.7
	Microphthalmia/ anophthalmia					0.0
Musculoskeletal	Spiral toe growth		8	16	24	16.4
	Hyperextension of the fetlock joint		11	7	18	12.3
	Angular limb deformities		1	15	16	11.0
	Axial rotation of the limbs		3	9	12	8.2
	Polydactyly		3		3	2.1
	Syndactyly					0.0
Reproduction	Supernumerary teats		2	8	10	6.8
	Cryptorchism	2	3	3	8	5.5
	Increased infertility			5	5	3.4
	Hermaphrotidism		2	1	3	2.1
	Atresia vulvi		1	1	2	1.4
Other	Crocked tail			8	8	5.5
	Hernia		1	3	4	2.7
	Atresia ani		2	1	3	2.1
Behaviour	Difficulties in coordination		1	3	4	2.7
	Tremor		2	1	3	2.1
	Paralysis	1	1		2	1.4
	Abnormal movements					0.0
	**Total Observations**	3	60	98	161	

n = number of farms
* blue-eyed white
